# Synergistic Antitumorigenic Activity of Calcitriol with Curcumin or Resveratrol is Mediated by Angiogenesis Inhibition in Triple Negative Breast Cancer Xenografts

**DOI:** 10.3390/cancers11111739

**Published:** 2019-11-06

**Authors:** Janice García-Quiroz, Rocío García-Becerra, Clara Santos-Cuevas, Gerardo J. Ramírez-Nava, Gabriela Morales-Guadarrama, Nohemí Cárdenas-Ochoa, Mariana Segovia-Mendoza, Heriberto Prado-Garcia, David Ordaz-Rosado, Euclides Avila, Andrea Olmos-Ortiz, Sofía López-Cisneros, Fernando Larrea, Lorenza Díaz

**Affiliations:** 1 Departamento de Biología de la Reproducción Dr. Carlos Gual Castro, Instituto Nacional de Ciencias Médicas y Nutrición Salvador Zubirán, Vasco de Quiroga No. 15, Belisario Domínguez Sección XVI, Tlálpan 14080, Ciudad de México, Mexicorocio.garciab@iibiomedicas.unam.mx (R.G.-B.); gabriela.mguadarrama@gmail.com (G.M.-G.); cardenas9501@gmail.com (N.C.-O.); davo21_05@hotmail.com (D.O.-R.); euclides.avilac@incmnsz.mx (E.A.); biol.dslc@gmail.com (S.L.-C.); fernando.larreag@gmail.com (F.L.); 2 Departamento de Biología Molecular y Biotecnología, Instituto de Investigaciones Biomédicas, Universidad Nacional Autónoma de México, Av. Universidad 3000, Coyoacán 04510, Ciudad de México, Mexico; 3 Departamento de Materiales Radiactivos, Instituto Nacional de Investigaciones Nucleares, Ocoyoacac 52750, Estado de México, Mexico; clara_letici@yahoo.com.mx (C.S.-C.); gerjul5420@hotmail.com (G.J.R.-N.); 4 Departamento de Inmunología, Instituto de Investigaciones Biomédicas, Universidad Nacional Autónoma de México, Av. Universidad 3000, Coyoacán 04510, Ciudad de México, Mexico; monaco445@yahoo.com.mx; 5 Departamento de Enfermedades Crónico-Degenerativas, Instituto Nacional de Enfermedades Respiratorias Ismael Cosío Villegas, Calzada de Tlalpan 4502, Belisario Domínguez Sección XVI, Tlalpan 14080, Ciudad de México, Mexico; hpradog@yahoo.com; 6 Departamento de Inmunobioquímica, Instituto Nacional de Perinatología Isidro Espinosa de los Reyes, Montes Urales 800, Lomas-Virreyes, Lomas de Chapultepec IV Sección, Miguel Hidalgo 11000, Ciudad de México, Mexico; nut.aolmos@gmail.com

**Keywords:** angiogenesis, breast cancer, calcitriol, curcumin, resveratrol

## Abstract

Calcitriol is a multitarget anticancer hormone; however, its effects on angiogenesis remain contradictory. Herein, we tested whether the antiangiogenic phytochemicals curcumin or resveratrol improved calcitriol antitumorigenic effects in vivo. Triple-negative breast cancer tumoral cells (MBCDF-T) were xenografted in nude mice, maintaining treatments for 3 weeks. Tumor onset, volume and microvessel density were significantly reduced in mice coadministered with calcitriol and curcumin (Cal+Cur). Vessel count was also reduced in mice simultaneously treated with calcitriol and resveratrol (Cal+Rsv). Cal+Cur and Cal+Rsv treatments resulted in less tumor activated endothelium, as demonstrated by decreased tumor uptake of integrin-targeted biosensors in vivo. The renal gene expression of *Cyp24a1* and *Cyp27b1* suggested increased calcitriol bioactivity in the combined regimens. In vitro, the phytochemicals inhibited both MBCDF-T and endothelial cells proliferation, while potentiated calcitriol’s ability to reduce MBCDF-T cell-growth and endothelial cells migration. Resveratrol induced endothelial cell death, as deduced by increased sub-G1 cells accumulation, explaining the reduced tumor vessel number in resveratrol-treated mice, which further diminished when combined with calcitriol. In conclusion, the concomitant administration of calcitriol with curcumin or resveratrol synergistically promoted anticancer effects in vitro and in vivo in human mammary tumor cells. Whereas the results suggest different mechanisms of action of the phytochemicals when coadministered with calcitriol, the converging biological effect was inhibition of tumor neoangiogenesis.

## 1. Introduction

A great body of evidence supports a positive correlation between microvascular density, tumor grade, and aggressiveness [1,2,3]. Indeed, in breast carcinoma and other neoplasias, the incidence of metastatic disease increases with the number of microvessels in the tumor mass [4], highlighting the importance to develop therapeutic strategies targeting angiogenesis. Calcitriol (1,25-dihydroxyvitamin D_3_), the vitamin D active metabolite, has been widely studied in pharmacological doses as a promising oncological drug based on its antitumoral properties [5,6,7]. Calcitriol biosynthesis begins in the skin, where the UVB radiation breaks the B ring of 7-dehydrocholesterol, resulting in cholecalciferol formation. This precursor suffers two hydroxylation reactions taking place first in the liver to obtain 25-hydroxyvitamin D_3_ (calcidiol), and second in the kidney and other tissues that express CYP27B1, the cytochrome that catalyzes the bioconversion of calcidiol into calcitriol. As a feedback regulatory mechanism, calcitriol inhibits the expression of CYP27B1 while induces that of CYP24A1, the cytochrome that degrades calcitriol into less biologically active metabolites. Several epidemiologic studies have demonstrated an inverse association between serum calcidiol levels and the risk to develop breast cancer [8]. The explanation of this association may reside in the increased vitamin D receptor (VDR) expression in breast cancer tumors of all phenotypes, making them good targets for calcitriol anticancer effects [9]. Indeed, calcitriol, via the VDR, inhibits cell proliferation, oncogenes expression and tumorigenesis through different mechanisms [6,10]. Moreover, calcitriol acts as an endogenous natural cancer-preventing factor due to its pro-differentiating properties [11]. However, the effects of calcitriol on angiogenesis are not yet conclusive, since opposite outcomes have been reported. In this regard, antiangiogenic calcitriol effects include its ability to reduce umbilical vein endothelial cells proliferation in vitro and to inhibit vessel formation in vivo [12,13]. On the contrary, substantial evidence has shown a calcitriol-dependent enhancement of the angiogenic potential on cancer and endothelial cells both in vitro and in vivo [14,15,16,17]. Particularly in breast cancer, the induction of a proangiogenic phenotype by calcitriol is supported by its ability to stimulate the expression of proangiogenic molecules such as vascular endothelial growth factor (VEGF), fibroblast growth factor (FGF), and cathelicidin, and to inhibit the angiogenesis inhibitor thrombospondin-1 [18,19], suggesting a differential effect of calcitriol depending on the tumor type. Additional preclinical evidence has shown that calcitriol and its analogues enhance tumor blood flow and perfusion [20]. Considering all the above, in this breast cancer study, we hypothesized that combining calcitriol with an anti-angiogenic factor would greatly improve calcitriol antineoplastic effects in vivo. We aimed at selecting natural compounds in order to increase the possibility to translate our findings into an adjuvant clinical therapy with no associated toxicity and economically affordable. Therefore, we selected the phytochemicals curcumin and resveratrol. Curcumin, derived from the plant *Curcuma longa*, is a diarylheptanoid nutraceutical with antiproliferative, proapoptotic, and antioxidant effects, which also exerts anti-tyrosine kinase activity [21,22]. Tyrosine kinase receptors (RTKs) play a critical role in carcinogenesis and mediate the effects of VEGF and FGFs, so in this study, the use of curcumin as RTK-inhibitor was intended. Resveratrol, on the other hand, is a natural polyphenol found in seeds and fruits, which interferes with signaling pathways involved in cell proliferation, apoptosis, and metastasis [23]. We chose these compounds for two main reasons: (1) Both curcumin and resveratrol have proven ability to obstruct angiogenesis [24,25] and (2) both significantly potentiate calcitriol actions by facilitating the heterodimerization of VDR with its partner retinoid X receptor (RXR), showing a cooperative effect on gene transactivation [26,27]. Furthermore, curcumin has been identified as a VDR ligand in human cancer cells [28] and the combination of calcitriol with curcumin has shown to inhibit leukemia cells proliferation to a greater extent than either compound alone [27,29]. Regarding resveratrol, clinical and preclinical studies have found that this phytochemical increases the bioavailability and plasma concentration of several drugs by inhibiting hepatic expression of CYP3A4 [30]. Considering that this cytochrome is also the major source of oxidative metabolism of calcitriol in the human liver [31,32], we speculated that CYP3A4 inhibition by resveratrol would represent an additional mechanism of the phytochemical to potentiate calcitriol antineoplastic effects. For this study, we chose a highly invasive human breast cancer cell line representing a clinically challenging tumor phenotype with few therapeutic options: the triple negative breast cancer (TNBC). Thus, herein we aimed at improving calcitriol antitumorigenic activity by combining it with the phytochemicals resveratrol or curcumin in order to reduce angiogenesis in mice xenotransplanted with human TNBC cells.

## 2. Results

### 2.1. Characterization of Cell Lines and Tumors

#### 2.1.1. MBCDF-T and EA.hy926 Cells Expressed VDR and RXR in Different Cell Compartments

**Figure 1:**
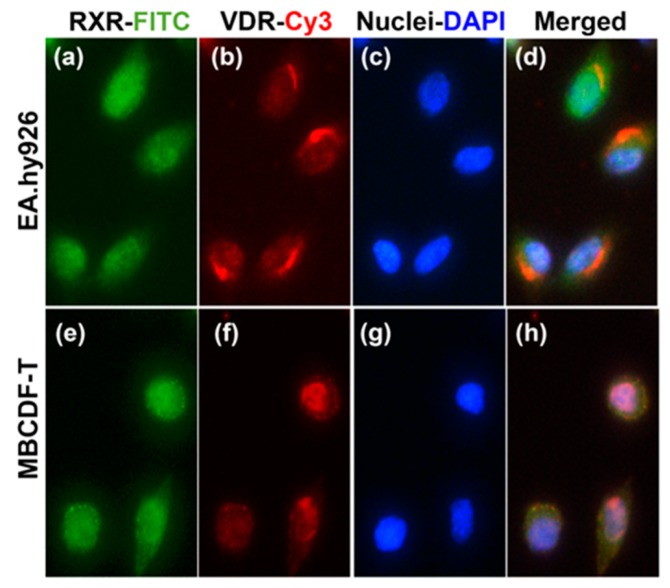
**Vitamin D receptor** (VDR) and retinoid X receptor (RXR) immunolocalization in EA.hy926 and MBCDF-T cells. EA.hy926 (**a**–**d**) or triple-negative breast cancer tumoral cells (MBCDF-T) (**e**–**h**) were seeded onto chamber slides. After fixing and permeabilizing, slides were co-incubated with rabbit anti-RXR and mouse anti-VDR antibodies. The secondary antibodies goat anti-rabbit-Fluorescein isothiocyanate (FITC) and goat anti-mouse-Cy3 allowed to identify RXR in green (**a**,**e**) and VDR in red (**b**,**f**). DAPI shows nuclei in blue (**c**,**g**). Merged photographs are shown in (**d**,**h**). Magnification: 40 ×).

For in vitro studies, we used two different cell lines that would help us understand the effects of the treatments in therapeutically relevant cell lineages: endothelial and tumoral (EA.hy926 and MBCDF-T, respectively). To ascertain whether these cells were able to respond to calcitriol, we first assessed VDR/RXR expression by immunocytochemistry. As seen in Figure 1, both EA.hy926 and MBCDF-T cells expressed the VDR and its hetero-partner RXR, which suggests their sensitivity to calcitriol. Interestingly, these transcription factors were found in different cell compartments depending on the cell type. Whereas both RXR and VDR were preferentially located in the nucleus of MBCDF-T cells, in EA.hy926 cells, RXR was found in the cytoplasm and nuclei, while VDR-protein appeared to be located in the Golgi complex and endoplasmic reticulum (Figure 1).

#### 2.1.2. MBCDF-T Tumors were ERα, PR, and HER2 Negative with a Mesenchymal-Like Phenotype

Immunohistochemistry showed that xenotransplanted MBCDF-T cells developed tumors that were negative for estrogen receptor (ER)α, progesterone receptor (PR), cytokeratin-7 (CK-7), and epidermal growth factor receptor 2 (HER2), but positive for vimentin (Supplementary Figure S1), representing a TNBC phenotype. The positive expression of vimentin, a marker of epithelial-to-mesenchymal transition (EMT) and basal-like breast cancer suggested that tumor cells have undergone EMT [33].

### 2.2. The Phytochemicals and Calcitriol Differentially Regulated the Proliferation of MBCDF-T and EA.hy926 Cells In Vitro

**Figure 2:**
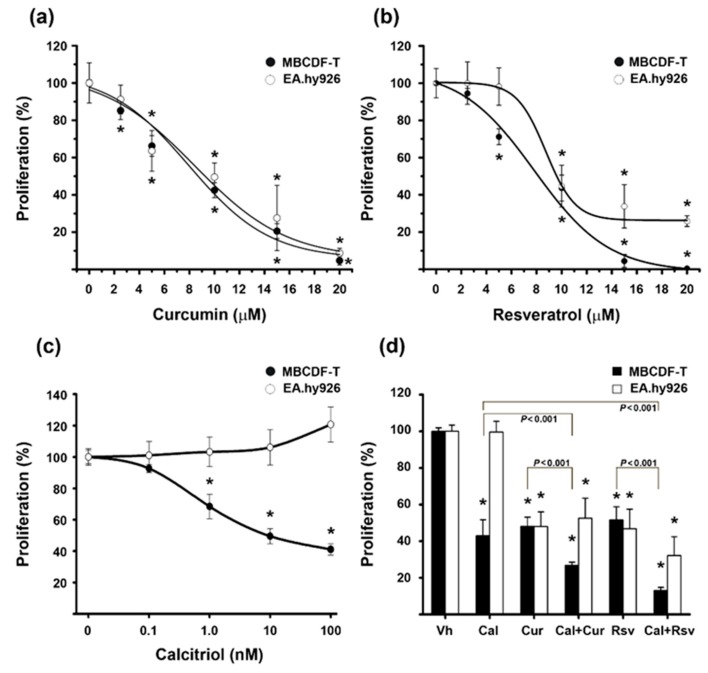
Effect of curcumin, resveratrol, and/or calcitriol in MBCDF-T and EA.hy926 cells proliferation. Dose-response curves of curcumin (**a**), resveratrol (**b**), and calcitriol (**c**) in MBCDF-T (black circles) and EA.hy926 cells (white circles). Medium containing vehicle (ethanol, 0.1%), calcitriol (0.1–100 nM), curcumin, or resveratrol (2.5–20 μM) was added in sextuplicate and incubated for 6 days. Curcumin and resveratrol inhibited cell proliferation in a dose-dependent manner in both MBCDF-T cells and EA.hy926, while calcitriol only decreased the proliferation of the MBCDF-T cells. (**d**) Drug combinations study. Incubation of MBCDF-T (black bars) and EA.hy926 (white bars) were performed during 6 days in the absence or the presence of calcitriol (Cal, 10 nM), curcumin (Cur, 10 μM), resveratrol (Rsv, 10 μM), or the combination of calcitriol with curcumin (Cal+Cur) or with resveratrol (Cal+Rsv). Results are depicted as the mean ± SEM of sextuplicate determinations in at least three different experiments and were normalized vs. control or vehicle (Vh) values, which were set to 100%. * *p* < 0.001 vs. control. In panel (**d**), lines depict the significance between co-treatments and monotherapy (*p* < 0.001). For the cell line MBCDF-T, * *p* ˂ 0.001 vs. vehicle and for the cell line EA.hy926 * *p* ≤ 0.002 vs. both Vh- and Cal-treated cells.

To investigate the effect of the compounds alone and combined on breast cancer and endothelium cells, we performed proliferation assays. While curcumin and resveratrol significantly reduced the proliferation of both MBCDF-T and EA.hy926 cells (Figure 2a,b), calcitriol only affected MBCDF-T cells (Figure 2c). Moreover, at a high concentration (100 nM), calcitriol increased endothelial cells proliferation, although not significantly (Figure 2c). Interestingly, the combination of calcitriol with the two phytochemicals reduced breast cancer cells proliferation in a greater extent than each compound alone (*p* < 0.001), an effect that was not observed in endothelial cells (Figure 2d).

Based on the range of drug concentrations tested, inhibitory concentrations values at 50% (IC_50_) were calculated and are shown in Table 1. As depicted, calcitriol elicited antiproliferative effects upon MBCDF-T cells in the nanomolar range, while both curcumin and resveratrol demonstrated equipotent micromolar inhibitory concentrations.

### 2.3. Depending on the Cell Line, the Experimental Compounds Differentially Regulated the Cell Cycle Profile; particularly, Resveratrol Significantly Induced Endothelial Cell Death

**Figure 3:**
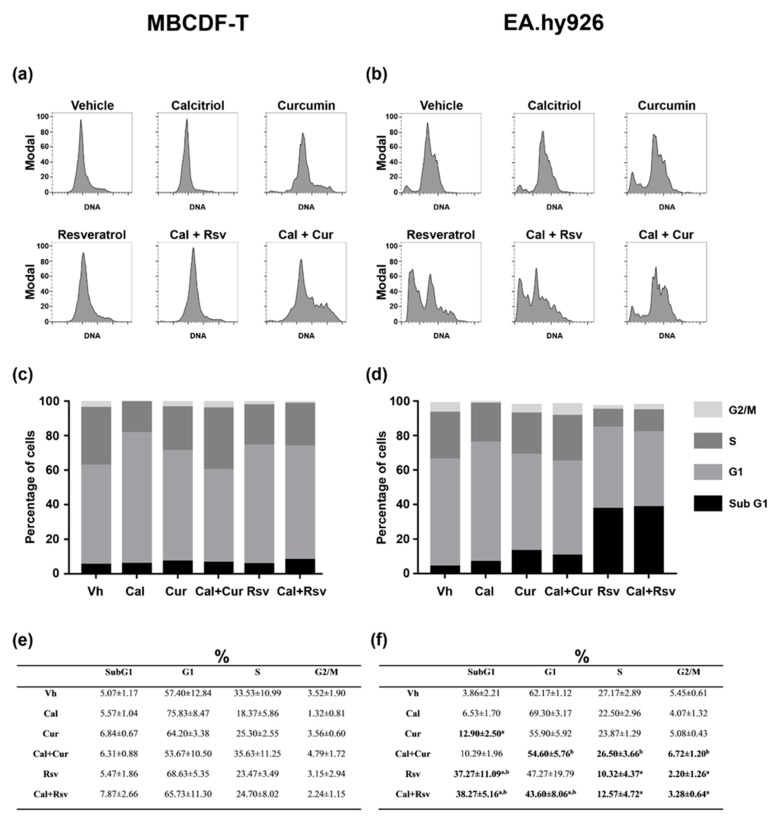
Effects of calcitriol, curcumin, resveratrol, and the combination of calcitriol with phytochemicals on the cell cycle. MBCDF-T (**a**,**c**,**e**) and EA.hy926 (**b**,**d**,**f**) cells were exposed to vehicle (Vh), calcitriol (Cal, 10 nM), curcumin (Cur, 10 μM), resveratrol (Rsv, 10 μM), or the combination of calcitriol plus curcumin (Cal+Cur) or resveratrol (Cal+Rsv) during 72 h and were analyzed by flow cytometry. Representative images per treatment are shown in (**a**,**b**). The percentage of cells in each cell cycle phase is depicted in the graphs (**c**,**d**) and in the tables (**e**,**f**). Results are shown as the percentage ± S.D. of cells per cell cycle phase from three independent assays. ^a^ *p* < 0.05 vs. vehicle-treated cells, ^b^ *p* < 0.05 vs. Cal (one-way ANOVA).

In order to gain mechanistic insights into the effects of the treatments on tumor and endothelial cells, we analyzed the cell cycle profile in cultured MBCDF-T and EA.hy926 cells after 72 h of exposure to calcitriol, curcumin, resveratrol, and the combination of calcitriol with the phytochemicals. As depicted in Figure 3, in MBCDF-T cells calcitriol arrested cells in the G1-phase (75.8% vs. 57.4% for calcitriol vs. vehicle treated cells, respectively), which was accompanied by a concomitant reduction in the proportion of cells in S-phase, compared to vehicle (Figure 3e). Curcumin and resveratrol tended to present a similar behavior than calcitriol in this cell line. However, when the cells were exposed to Cal+Cur, the population in G1-phase was reduced while the percentage of cells in S-phase increased compared to either compound alone (Figure 3). On the other hand, the cell cycle profile of cells treated with Cal+Rsv was similar to that observed with each compound alone (Figure 3). Regarding EA.hy926 cells, and in a similar manner as in MBCDF-T cells, calcitriol tended to increase the percentage of cells in G1-phase (Figure 3). Curcumin, on the other hand, significantly induced cell death in a low proportion of endothelial cells (13%, identified as SubG1 cells), which was accompanied by a modest reduction in the G1-phase population. Remarkably, in EA.hy926 cellss, resveratrol induced a higher percentage of cells accumulated in SubG1 phase (37.27% vs. 3.86% for resveratrol vs. vehicle-treated cells, respectively), and when compared to vehicle, there was a significant reduction in all cell cycle phases. The combination Cal+Cur or Cal+Rsv provoked a similar effect on the cell cycle profile than that obtained with the phytochemicals alone (Figure 3b,d). Indeed, in endothelial cells, calcitriol did not significantly modify the effect of the phytochemicals on the cell cycle profile (Figure 3b,d).

### 2.4. The In Vivo Combination of Calcitriol and Curcumin Reduced the Number and Size of Breast Cancer Tumors

**Figure 4:**
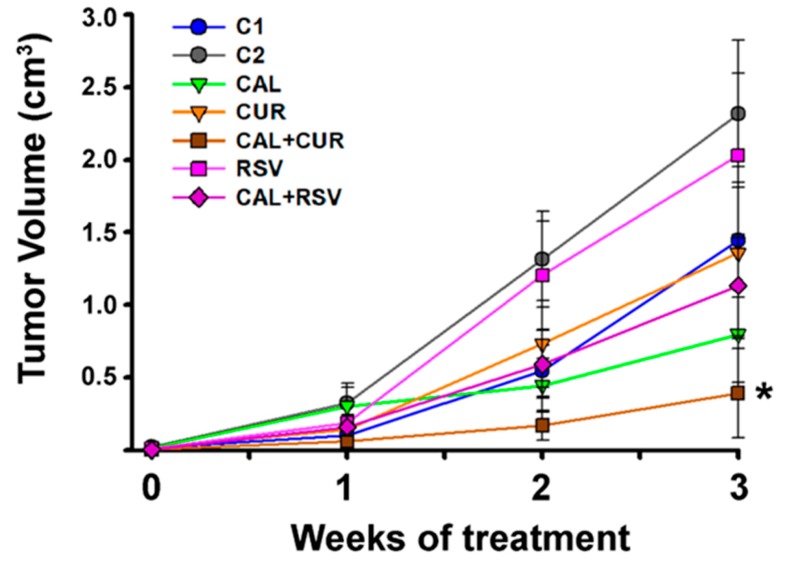
In vivo effect of calcitriol, curcumin, resveratrol, or the co-treatments in tumor volume. MBCDF-T cells were xenografted in female nude mice. Treatments started the following day and lasted 3 weeks. Mice were treated with saline solution (C1), vehicle (C2), calcitriol (Cal), curcumin (Cur), resveratrol (Rsv), or the combination of calcitriol with resveratrol (Cal+Rsv) or with curcumin (Cal+Cur). * *p* < 0.05 vs. C1 and C2. *N* ≥ 9, mean ± SEM.

MBCDF-T cells resulted highly tumorigenic, since 94% and 100% of mice xenotransplanted with this cell line and treated either with saline (C1) or vehicle (ethanol 0.1%, C2), respectively, developed tumors. Mice that received resveratrol, alone and combined, or curcumin alone, also generated tumors in all cases. Importantly, in calcitriol-treated mice and Cal+Cur groups, only 83.3% and 58% of animals, respectively, generated tumors at the end of the experiment, indicating that these treatments delayed tumor onset. After 3 weeks of therapy, the mice in the calcitriol group displayed smaller tumors than those in the control groups C1 and C2, while curcumin alone also reduced tumor volume when compared to its vehicle (C2), although not significantly (Figure 4). However, the combination of calcitriol with curcumin significantly reduced tumor volume in comparison with all other groups. Meanwhile, the groups with the largest tumors were the two controls and resveratrol alone (Figure 4). Noteworthy, no signs of treatment-related toxicity were detected (dehydration, changes in overall appearance and activity or significant weight loss). In this regard, all mice receiving calcitriol were generally leaner than their counterparts; however, total body weight was not significantly different between groups at the end of the experiments (mean 26.4 g ± 1.0 g considering all groups).

### 2.5. Tumors in the Cal+Cur and Cal+Rsv Groups Had a Reduced Intratumor Angiogenic Network Compared to Controls

**Figure 5:**
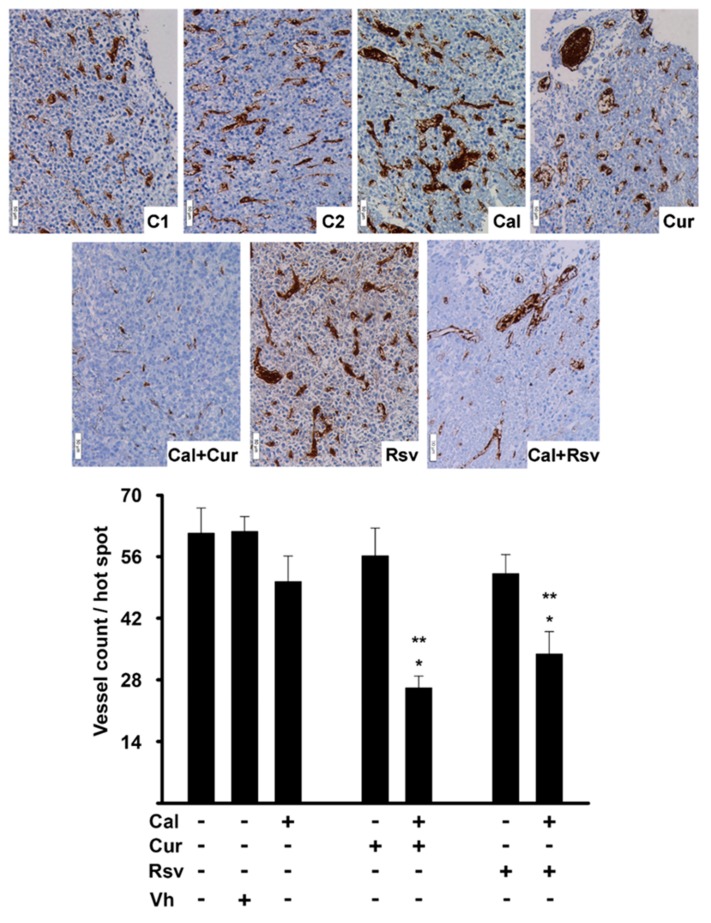
In vivo, the co-treatment of calcitriol with curcumin or resveratrol reduced vessel density and diameter. Itgb3 immunohistochemistry (brown staining) was used to identify and count vessels in the tumor tissue of mice treated with: saline solution (C1), vehicle (C2), calcitriol (Cal), curcumin (Cur), resveratrol (Rsv), or the co-treatment of calcitriol with curcumin or resveratrol (Cal+Cur or Cal+Rsv, respectively). Representative images are shown in the upper panel; magnification 20×, scale bar indicates 50 μm. In the lower panel, vessel count results are depicted as the mean ± SEM. Three hot spots per tumor were counted in at least 10 different tumors per treatment by three different observers. * *p* < 0.001 vs. control groups, ** *p* < 0.05 vs. each compound alone.

In order to determine if the observed reduced tumor volume in the Cal+Cur group was related to differences in the intratumor vessel network, microvessel count was performed using the integrin subunit β (Itgb3) as a marker of activated endothelium. In fact, this integrin was expressed only by endothelial cells in the tumor vessels (Figure 5). As expected, Cal+Cur was the group that displayed the fewest number of intratumor microvessels (*p* < 0.05), which also had the smallest vessel diameters (Figure 5). Remarkably, despite the large mean tumor size in the resveratrol group, the combination of calcitriol with this compound also showed a significantly reduced number of vessels per hot spot in the tumor cross sections (Figure 5). This effect was not expected, given that when excising the tumors of resveratrol-treated mice, dilated blood vessels and blood lacunas were found neighboring the tumor perimeter. Of note, the tumors of mice in C1, C2, calcitriol, curcumin, and resveratrol treated groups showed a highly disorganized vascular network, with a pattern consisting of structural abnormalities characterized by dilatation of vessel diameter, hyper-branching, and loss of vessel hierarchy (Figure 5). On the contrary, tumor vessel diameter was consistently reduced in Cal+Cur and Cal+Rsv groups, with a morphology suggesting vascular normalization (Figure 5).

### 2.6. Tumor Uptake of Integrin-Targeted Biosensors Was Proportional to the Tumor Size

**Figure 6:**
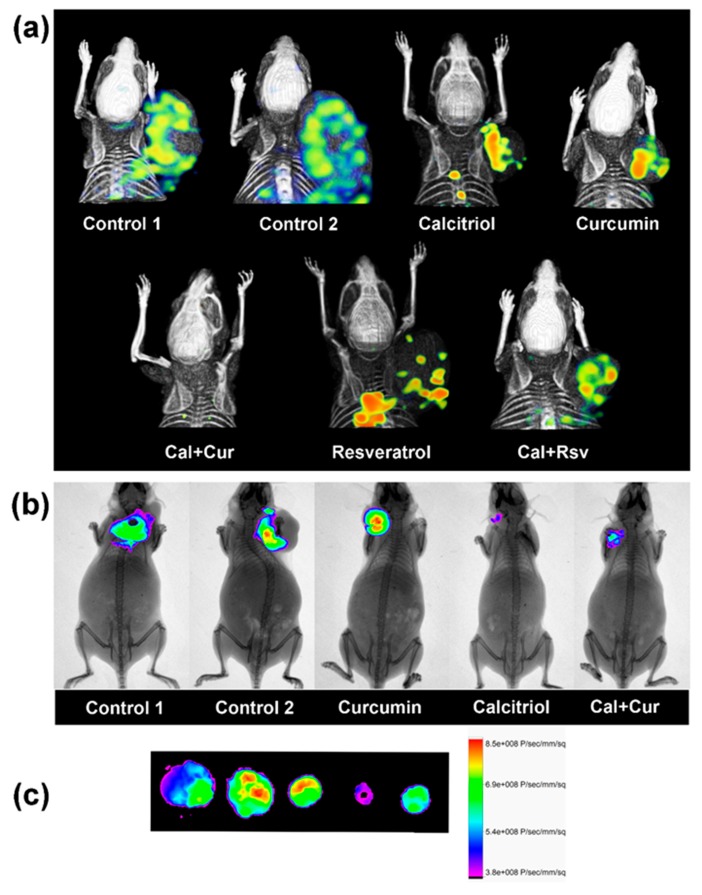
Tumor uptake of integrin-targeted biosensors was proportional to the tumor size. (**a**) In vivo ^99m^Tc-RGD_2_ tumor uptake in representative mice of each experimental group was evaluated using a micro-SPECT/CT scanner. Isoflurane-anesthetized mice were intravenously injected with ^99m^Tc-RGD_2_ and images were acquired 3–4 h after the administration. (**b**) Fluorescence and X-ray images were acquired with the optical/X-ray Imaging System In-Vivo Xtreme to verify tumor uptake 48 h after the intravenous injection of IntegriSense™680. (**c**) Ex vivo tumor distribution of the fluorescent probe. After excising the tumors, the distribution of the fluorescent dye was assessed to complete the in vivo analysis. From left to right, tumor order is the same as in (**b**). Representative images are shown. The co-treatment of calcitriol with curcumin or resveratrol is depicted as Cal+Cur or Cal+Rsv, respectively.

The noncovalently-bound integrin subunits αv (Itgav) and Itgb3, namely vitronectin receptor (αvβ3), was used as target to study tumor angiogenesis in vivo, given that αvβ3 is highly expressed on activated endothelial and tumor cells [34]. Therefore, we explored tumor uptake of two different αvβ3-targeting pharmaceutical compounds: ^99m^Tc-RGD_2_ and IntegriSense™680. We found that tumor uptake of these active-endothelium-targeted bioprobes was directly proportional to the tumor size, suggesting less integrin abundance in the calcitriol-treated groups compared to controls; specially in the Cal+Cur group (Figure 6).

It should be noted that tumors in C1, C2, and resveratrol groups grew very rapidly, and at the end of the experiment wide areas of necrosis were observed, which probably explained the lack of homogeneity in the biosensors intratumor uptake. Moreover, in these tumors, ^99m^Tc-RGD_2_ images suggested new spots of tumor colonization away from the original tumor mass, which were not appreciated externally and may represent metastasis (Figure 6a). In contrast, in calcitriol, curcumin, Cal+Cur, and Cal+Rsv groups, there were smaller tumors that grew slowly and, at the time of image acquirement, were suggestive of young developing tumors with active endothelium areas, probably representing newborn microvessels (Figure 6a). Therefore, we decided to use a different integrin-based analysis to ascertain results. Since treatment with curcumin and its combination with calcitriol showed better results, only these groups were considered for fluorescence imaging. As depicted in Figure 6b, IntegriSense™680 distribution in vivo indicated that the tumors of C1, C2, and curcumin-treated mice had similar affinity for the molecular fluorescent dye; however, calcitriol and Cal+Cur administration significantly reduced IntegriSense™680 tumor uptake, suggesting that ανβ3-dependent signaling decreased compared to control mice. This was further confirmed by ex vivo imaging (Figure 6c) and was consistent with the observed tumor volume.

### 2.7. In Vivo Bioactivity of the Experimental Compounds Was Corroborated by the Modification of Renal Cyp24a1 and Cyp2b1, and Hepatic Cyp3a44 and GPx1 Gene Expression

**Figure 7:**
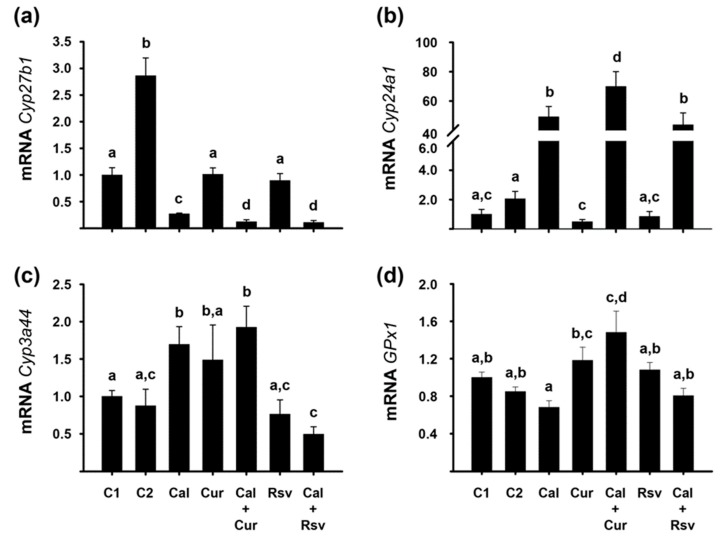
Curcumin and resveratrol enhanced the bioactivity of calcitriol, as deduced by the gene expression profile of vitamin D-metabolic cytochromes in the kidney of treated mice, while the phytochemicals biological effect was reflected in the liver biomarkers *GPx1* and *Cyp3a44*. (**a**,**b**) Renal mRNA expression of *Cyp27b1* and *Cyp24a1*. (**c**,**d**) Hepatic mRNA expression of *Cyp3a44* and *GPx1*. Mice were treated for three weeks with saline solution (C1), vehicle (C2), calcitriol (Cal), curcumin (Cur), resveratrol (Rsv), or the co-treatment of calcitriol with Cur or Rsv (Cal+Cur or Cal+Rsv, respectively). The RT-qPCR results were normalized against the gene expression of *Actb* and vs. C1 values, which were set to 1. Mean ± SEM. Bars not sharing a letter differ significantly (*p* < 0.05). *N* ≥ 9.

We used different parameters to evaluate the bioactivity of calcitriol and the phytochemicals. For calcitriol, we assessed the gene expression of *Cyp27b1* and *Cyp24a1*, which encode for vitamin D activating and catabolizing enzymes, respectively. These two genes are inversely regulated by calcitriol in the kidney as a feed-back regulatory loop. In our study and as expected, calcitriol-treated mice had significantly lower *Cyp27b1* and higher *Cyp24a1* renal gene expression (Figure 7a,b), a sign of elevated systemic calcitriol concentrations. Interestingly, the co-administration of calcitriol with both phytochemicals further reduced *Cyp27b*1 gene expression compared to calcitriol alone (*p* < 0.05, Figure 7a), while Cal+Cur treatment upregulated *Cyp24a1* gene expression in a greater extent than the secosteroid hormone per se (Figure 7b), overall suggesting increased calcitriol bioactivity. An unexpected result was the upregulation of *Cyp27b1* in the C2 group (*p* < 0.05).

To ascertain resveratrol and curcumin bioactivity, we studied the gene expression of *Cyp3a44* and *GPx1* in the liver. In this regard, the inhibitory effect of resveratrol on *Cyp3a4* in vitro and in vivo has been well established [30], while calcitriol transcriptionally stimulates this gene due to a functional vitamin D responsive element (VDRE) in its promoter [35]. Herein, the *Cyp3a4* isoform that was amplified was the one encoded by the *Cyp3a44* gene, given that in the female mice liver, this is one of the predominant cytochromes involved in the oxidative metabolism of endogenous and exogenous compounds [36]. Herein, resveratrol downregulated *Cyp3a44* gene expression per se; although not significantly. However, a significant downregulation of *Cyp3a44* gene expression was observed in the Cal+Rsv group. As expected, *Cyp3a44* gene expression was significantly stimulated by calcitriol and Cal+Cur treatments, while it was reduced by Cal+Rsv, compared to their controls (Figure 7c). On the other hand, it has been shown that *GPx1* is transcriptionally upregulated by curcumin in the liver of curcumin-treated mice [37]. Accordingly, in this study, hepatic *GPx1* gene expression was slightly upregulated by curcumin alone, an effect that was significantly enhanced by the cotreatment with Cal+Cur, compared to the controls (Figure 7d).

### 2.8. Curcumin Combined with Calcitriol Was the Most Efficient Strategy to Prevent Endothelial Cells Migration

**Figure 8:**
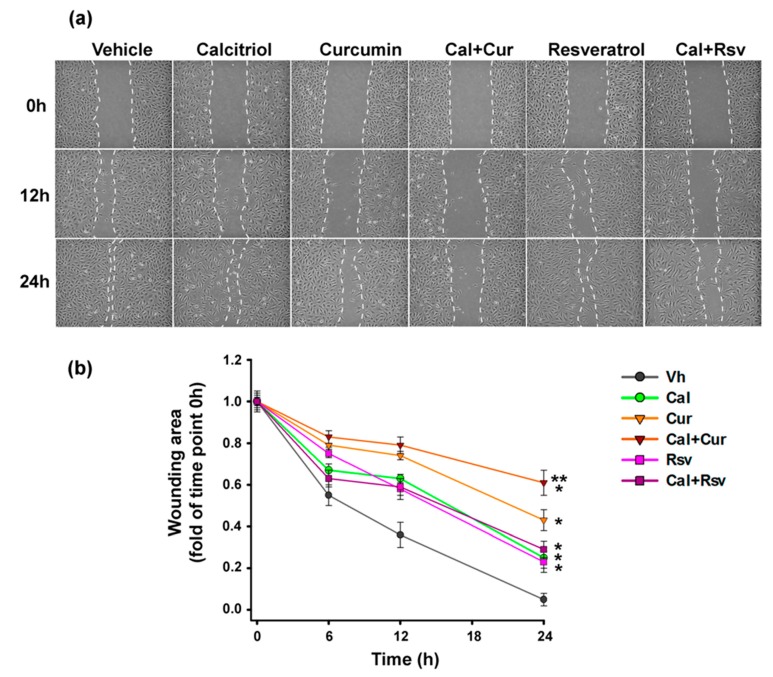
The combination of Calcitriol+Curcumin inhibits in vitro the migratory activity of EA.hy926 cells. (**a**) Representative bright-field images (10×) showing the effects of calcitriol (Cal, 10 nM), curcumin (Cur, 10 μM), resveratrol (Rsv, 10 μM), or the combinations of calcitriol with curcumin (Cal+Cur) or with resveratrol (Cal+Rsv). The profile of the wound is delineated for better visualization. (**b**) Wound closure expressed as the remaining area uncovered by the cells. The scratch area at time point 0 h was set to 1. Scratch-wound closure was monitored over time (0, 6, 12, and 24 h) in triplicate experiments, *N* = 9 measures per treatment; * *p* < 0.05 vs. vehicle-treated cells (ethanol 0.1%), ** *p* < 0.05 vs. calcitriol or curcumin alone.

Given that metastasis to distant sites depends on the ability of cancer cells to enter the circulation, preventing tumor-associated angiogenesis is a crucial opportunity to block this process. Therefore, to study the effect of the treatments upon endothelial cells migration, herein we used the wound healing assay. As seen in Figure 8, all treatments tested resulted in a lower migration speed compared to vehicle-treated cells (*p* < 0.05). However, the combination of calcitriol plus curcumin decreased, in a greater extent, the closure of the wounding area compared to either treatment alone (Figure 8).

## 3. Discussion

Triple negative breast cancer remains a challenging clinical entity due to its highly metastatic/proliferative nature and the absence of ER, PR, and HER2 expression, which desensitize it for hormonal and anti-HER2 therapies. To date, no suitable targeted-therapy is available for patients with TNBC. Efforts in research and development of novel drug combinations could certainly help to improve treatment options for this tumor phenotype. Calcitriol is a multitarget anticancer hormone with proven ability to inhibit TNBC cells proliferation; however, it does not consistently restrain angiogenesis in breast tumors. In this study, our ultimate goal was to propose the use of natural compounds to synergize calcitriol’s antineoplastic effects in TNBC in a clinically relevant manner by targeting tumor angiogenesis and increasing its antiproliferative activity, without undesired side effects and with the additional benefit of being affordable. 

### 3.1. The Phytochemicals Curcumin and Resveratrol Enhanced the Antineoplastic In Vivo Effect of Calcitriol by Inhibiting the Adequate Formation of a Tumor Vessel Network 

Herein, we show for the first time that the in vivo antineoplastic effect of calcitriol was significantly improved by its combination with curcumin or resveratrol in TNBC. Calcitriol and the phytochemicals elicited differential effects upon several parameters depending on the cell line and experimental setting. In vivo, Cal+Cur treatment significantly reduced tumor onset and volume, Itgb3 expression, microvessel count, and tumor uptake of integrin-targeted biosensors, strongly suggesting an antiangiogenic promoting effect of the drug combination. Resveratrol, on the other hand, did not increase calcitriol’s ability to inhibit tumor growth or ^99m^Tc-RGD_2_ uptake, but significantly induced endothelial cell death, as determined in vitro by flow cytometry results, which probably explains the reduced number of tumor microvessels found in the Cal+Rsv group. The latter suggests that calcitriol potentiated in vivo the antiangiogenic effect of resveratrol, since at the dose tested the two compounds alone were not able to inhibit angiogenesis. 

### 3.2. The Differential Renal and Hepatic Expression of P450 Cythochromes and GPx1 among the Experimental Groups Reflected the Compounds Interaction and Bioactivity In Vivo 

The supposition that calcitriol enhanced resveratrol’s antiangiogenic activity is supported by the significant downregulation of *Cyp3a44* gene expression observed only in the liver of Cal+Rsv-treated mice. Indeed, the inhibition of this cytochrome expression by resveratrol has been previously reported [30,38] and was used herein as a positive control for resveratrol bioactivity. Considering this, the fact that in this study resveratrol alone did not affect *Cyp3a44* gene expression or tumor volume, could be attributed to the low in vivo dose administered. In fact, resveratrol was administered only three times a week orally, while curcumin was given ad libitum in the drinking water during the whole experiment. In this regard, low bioavailability of curcumin and resveratrol has generally been a concern due to poor solubility, low absorption, and rapid metabolism [39]. However, as judged by our results, the solubilization and dilution procedures, as well as the constant supply of curcumin used in this research, were enough to efficiently synergize calcitriol antitumorigenic effects and to evoke antioxidant activity, as depicted by the reduced tumor growth and upregulation of liver *GPx1* gene expression, respectively. Regarding GPx1, the antioxidant effect of this enzyme is of paramount importance in cancer treatment, given that reactive oxygen species (ROS) promote cancer progression, as has been demonstrated by decreased tumor recurrence and metastasis markers by ROS scavenging [40,41], as well as by the induction of cancer cells death and tumor shrinkage by *GPx1* overexpression and curcumin administration in breast cancer and lymphoma [37,42,43]. Therefore, it is possible that this antioxidant pathway partially contributed to the antitumorigenic effect of curcumin observed herein. Alternatively and/or concomitantly, a synergistic pro-differentiating mechanism might also be taking place in the Cal+Cur combination, in a similar manner as shown previously in leukemia cells [27,29]. Of note, the ability of vitamin D compounds to inhibit cancer stem-like cells and induce differentiation has been demonstrated in TNBC [11]. In our study, additional evidence on the ability of curcumin to synergize the effects of calcitriol is provided by the fact that renal gene expression of *Cyp27b1* and *Cyp24a1* was further inhibited and stimulated, respectively, in the Cal+Cur group, in comparison with calcitriol alone. This suggests a cooperative effect of curcumin and calcitriol on VDR transactivation, as shown elsewhere [27]. An unexpected result was renal *Cyp27b1* gene expression upregulation by the vehicle (C2), which we believe might reflect reduced systemic calcitriol levels, consistent with the increased tumor volume observed in this group. Supporting this, moderate ethanol intake has shown to decrease renal and tumoral 25-hydroxyvitamin D_3_ bioconversion into calcitriol [44]. Further investigation is required to know if alcohol administration on a daily basis at the concentrations used in this study modify vitamin D metabolism. 

### 3.3. In Vivo, the Combination of Cal+Cur Prevented Tumor Unset and Progression by Blocking Integrin Signalization in Both Tumor and Endothelial Cells, in which their Migratory Capacity Was also Blocked

We believe that for the Cal+Cur group, results in Figure 5 and Figure 6 correlate well given that the techniques used are based on detecting activated endothelium, which we found reduced by both methods, strongly suggesting a diminished neoangiogenic process. Indeed, considering that tumors in the Cal+Cur group showed reduced Itgb3 protein expression and little integrin αvβ3-targeted biosensors uptake, we deduced that one of the most relevant antitumorigenic effects of curcumin was the inhibition of neoangiogenesis. This was reinforced by previous findings showing that curcumin downregulated αvβ3 expression in other cancers [45]. Moreover, in MCF-7 breast cancer cells, calcitriol has shown to inhibit *Itgb3* gene expression [46]. The fact that in this study we found less Itgb3 protein expression in the tumors treated with the combinatorial regimens is of clinical relevance, since it is known that integrin signalization regulates tumor cells migration and invasion and that the expression of certain integrins correlates with increased disease progression and decreased patient survival [47]. Itgb3 is one of the two integrins that forms the αvβ3 receptor in endothelial cells, which plays a crucial role in tumor angiogenesis as inferred from studies showing that targeting αvβ3 resulted in a promising outcome in phase III clinical cancer trials [48]. Therefore, it is reasonable to assume that integrin inhibition by the combinatorial treatments prevented tumor progression by blocking critical signalization events in both the tumor microenvironment (including the host endothelial cells) and the tumor cells themselves. Remarkably, almost half of the Cal+Cur-treated mice did not generate tumors (42%), strongly suggesting an early operating tumor-preventing effect of the compound combination and therefore, the feasibility of its use for cancer recurrence and metastasis prevention. In this regard, and considering that tumor-associated angiogenesis is a critical step during the metastatic process, our results showing a decreased migration rate in endothelial cells treated with Cal+Cur further support the ability of this compounds combination to inhibit tumor nascent neovascularization and later metastasis. 

On the other hand, results in Figure 5 and Figure 6 for Cal+Rsv are a bit puzzling, since the combination of these compounds resulted in poorly vascularized tumors, as seen by immunohistochemistry, but still a relative active ^99m^Tc-RGD_2_ tumor uptake could be observed by micro-SPECT/CT scanner. We believe that the latter might be explained by the dilated blood vessels and lacunas that we found in the outer part of the tumors in resveratrol-treated mice, as described in Section 2.5, even though the tumor core showed a reduced vessel count. Another explanation may reside in the techniques used, since immunohistochemistry was performed using an antibody against only one integrin: Itgb3, while ^99m^Tc-RGD_2_ targets the vitronectin receptor, which is formed by both Itgav and Itgb3 integrins. It is possible that some tumor cells expressing Itgav are capable of binding to the integrin-targeted radiotracer.

Although we did not measure curcumin serum levels, in a murine study of glioblastoma xenografts the intraperitoneal (i.p.) administration of curcumin significantly inhibited angiogenesis by achieving a peak plasma level of ~6 μM [49], which is in the range of concentrations that inhibited endothelial and cancer cell proliferation in our in vitro model. This allows us to infer that the in vivo administration method applied in this research may have elevated curcumin serum levels within the micromolar range. 

### 3.4. In Vitro Mechanistic Insights and Clinical Implications

In vitro, the three experimental compounds significantly downregulated cancer cell proliferation, while the combination of calcitriol with either curcumin or resveratrol resulted in a greater reduction of cell growth. Notably, in an opposite manner as the phytochemicals, calcitriol did not reduce endothelial cell growth, explaining the lack of interaction between this hormone and the plant-derived compounds on EA.hy926 cell proliferation. The latter is consistent with flow cytometry results, which showed that endothelial cell death is not promoted by calcitriol. To date, contradictory outcomes have been reported regarding the effects of calcitriol on endothelial cells. Our results support and enlarge the existing evidence showing that in the context of breast cancer, calcitriol is not able to inhibit angiogenesis. Consequently, we advise the concomitant use of an antiangiogenic compound when administering calcitriol in adjuvant breast cancer treatment. Regarding the opposite effect of calcitriol upon the proliferation of cancer and endothelial cells, it is likely that this differential effect might be due to the distinct intracellular localization of the VDR in the two cell lines. Indeed, a significant antiproliferative effect was produced by calcitriol when its receptor was located into the nucleus of cancer cells, while no effect was produced in endothelial cells where the VDR resided in the endoplasmic reticulum. This particular location of the VDR has been reported in other cell types [50,51], where VDR is found in association with calreticulin [50]. Calreticulin is a calcium-sequestering protein with a known ability to block VDR signal transduction by interacting with its DNA binding domain [52], which may explain the lack of antiproliferative activity of calcitriol observed in our cultured endothelial cells and in tumor endothelium. Since the calcitriol-dependent increase in intracellular calcium levels has been associated to a cytoplasmic location of the VDR [51], further research is needed to investigate non-genomic rapid actions of calcitriol in endothelial cells and its relationship with cell proliferation. 

### 3.5. Highlights and Limitations of This Study

One limitation of our study was the low dose of resveratrol used in vivo; thus, more research is needed by testing it at a higher dose or using it on a daily basis. In addition, more experiments aimed at elucidating the signaling pathways implicated in the combination regimens are still needed.

Overall, the results generated herein strongly support that calcitriol anticancer effects were enhanced by its concomitant administration with curcumin. Data in this study also suggest that resveratrol is a good option, due to its ability to induce death in endothelial cells, which was improved by calcitriol. 

In summary, this study provides in vitro and in vivo evidence for the antitumorigenic synergistic effect of calcitriol with curcumin or resveratrol. Based on our results, we conclude that there is a dual anticancer effect of the phytochemicals when combined with calcitriol: (1) They inhibit tumor angiogenesis and (2) they boost calcitriol antiproliferative and pro-differentiating activity. Whereas the results suggest different mechanism of action of the phytochemicals, the shared elicited biological effects in the combination regimens included the reduction of breast cancer cell proliferation, tumor vessel number and diameter, as well as the downregulation of Itgb3 integrin expression. Thus, the combined strategy in adjuvancy may have the potential to overcome some major limitations of conventional chemotherapy in TNBC.

## 4. Materials and Methods 

### 4.1. Reagents

Curcumin, resveratrol, calcitriol, sulphorhodamine B (SRB), and trichloroacetic acid (TCA) for in vitro studies were from Sigma (Sigma–Aldrich, St Louis, MO, USA). For in vivo administration in mice, we used calcitriol and resveratrol gelcaps (Geldex^®^ and Veratrol^®^, respectively), both from GELpharma, México, while curcumin (Sigma) was first dissolved in ethanol and then diluted in drinking sterile water (total ethanol content in the drinking water was 0.1%). Curcumin dose was calculated considering a daily mouse drinking water intake of 5–7 mL [53]. IntegriSense™680 was from Perkin Elmer (Perkin Elmer, Waltham, MA, USA). The ^99m^Tc-RGD_2_ was obtained from Instituto Nacional de Investigaciones Nucleares (Estado de México, México).

### 4.2. Ethical Approval

Animals were handled following the rules and regulations of the Official Mexican Standard NOM-062-ZOO-1999. This study was approved by the Institutional Committee for the care and use of laboratory animals (protocol reference CINVA-1820 BRE-1820-16/19-1) of the Instituto Nacional de Ciencias Médicas y Nutrición Salvador Zubirán. Care was taken to minimize animal suffering by choosing the earliest endpoint compatible with the scientific objectives of this work, as described in detail previously [18].

### 4.3. Cell Culture

In this study, we used MBCDF cells kindly donated by María de Jesús Ibarra-Sánchez and Jesús Esparza-López (Instituto Nacional de Ciencias Médicas and Nutrición Salvador Zubirán, México). The MBCDF cell line was derived from a primary breast cancer cell culture generated from explants obtained from a radical mastectomy of a patient diagnosed with ductal infiltrating carcinoma stage IV with bone metastasis [54,55]. Herein, the parental MBCDF cell line was implanted in a mouse and the cell line derived from the resulting tumor was thereafter named MBCDF-Tum (MBCDF-T), which was used herein for all studies. For the in vitro proliferation studies, we additionally worked with a human umbilical vein endothelial cell line: EA.hy926 (ATCC CRL-292), kindly donated by Dr. Alejandro Zentella-Dehesa (Instituto de Investigaciones Biomédicas, UNAM, México). Cells were maintained in DMEM-F12 medium supplemented with 100 units/mL penicillin plus 100 μg/mL streptomycin and 5% heat-inactivated fetal bovine serum (FBS). All experimental procedures were carried out with supplemented medium using charcoal-stripped-FBS. 

### 4.4. Proliferation Studies

The ability of the different compounds to inhibit cell proliferation in vitro was studied by the sulphorhodamine (SRB) assay. Briefly: One thousand EA.hy926 cells or 500 MBCDF-T cells were plated in 96-well plates and incubated for 24 h. Afterwards, medium containing vehicle (ethanol, 0.1%), calcitriol (0.1–100 nM), curcumin, or resveratrol (2.5–20 μM) was added in sextuplicate and incubated for 6 days. Cells were fixed in ice cold TCA at 4 °C and air-dried. SRB solution was added to each well for 1 h and plates were washed with 1% v/v acetic acid to remove unbound dye. Solubilization of bound SRB was done by adding 10 mM tris base (pH 10.5) and shaking. Absorbance was determined at 492 nm in a microplate reader (Synergy™ HT Multi-Mode Microplate Reader, BioTek, VT, USA). The concentrations that caused 50% inhibition of cell growth (IC_50_) were determined using dose-effect curves of each compound to generate fitted curves by using the software Origin 8.0 (OriginLab Corporation, Northampton, MA, USA).

### 4.5. Cell Cycle Analysis

To define the effects of calcitriol alone and combined with the phytochemicals in the cell cycle distribution of cancer and endothelial cells, flow cytometric analyses were performed in cultured MBCDF-T and EA.hy926 cells. In brief, cells were treated with calcitriol (10 nM) with or without 10 μM curcumin or resveratrol during 72 h. After that, the cells were harvested, washed in PBS pH 7.2, fixed in 70% ethanol, and kept at −20 °C. The samples were washed twice with PBS and incubated in 0.5% v/v triton X-100 and DAPI (1 μg/mL) in the dark at room temperature for 20 min. The DNA content was determined using a FACS Aria II flow cytometer (Becton Dickinson, San Jose, CA, USA). For cell cycle analysis and SubG0 peak evaluations, a total of 20,000 events from DAPI-area vs. DAPI-wide gate were acquired. The results were analyzed using FlowJo Software (LLC, Ashland, OR, USA). 

### 4.6. Xenotransplantation and Therapeutic Protocol

Female athymic 6 weeks aged nude mice (BALB/c homozygous, Crl:NU(NCr)-Foxn1nu, Charles River Laboratories, Wilmington, MA, USA), were maintained under controlled temperature, humidity, and 12:12 light:dark periods with food (standard PMI 5053) and sterile water ad libitum. For the xenograft assay, MBCDF-T cells (1.0 × 10^6^/0.1 mL sterile 0.9% NaCl) were subcutaneously injected into the back of the mice and therapeutic treatments initiated the following day to target tumor neoangiogenesis from the beginning. Seven experimental groups were included: (1) Control (C1, Sterile saline 0.9% NaCl i.p. once a week), (2) Vehicle (C2, Ethanol 0.1% vol/vol in drinking water throughout the experiment), (3) Calcitriol (0.25 μg in 100 μL i.p. once a week), (4) Curcumin (40 mg/kg daily in drinking water throughout the experiment), (5) Resveratrol (1.2 g/kg orally three times a week), (6) Calcitriol + Curcumin (Cal+Cur), and (7) Calcitriol + Resveratrol (Cal+Rsv). Monotherapy and combinatorial treatments were maintained for 3 weeks. In the groups of curcumin and its vehicle (C2), the supplemented water was freshly prepared and replaced every day. Mice were weighed three times per week. A weight loss greater than 15% was considered a sign of toxicity. A caliper was used to assess tumor growth thrice weekly and tumor volume was calculated according to the standard formula (length × width^2^)/2. At the end of the experiments, mice were sacrificed by cervical dislocation under anesthesia (200 mg/kg i.p. of sodium pentobarbital) and tissues including tumor, kidney, and liver were collected and stored at −70 °C for RNA extraction. A portion of the tumor was fixed in paraformaldehyde for immunohistochemical studies.

### 4.7. In Vivo Imaging Studies of Activated Endothelium

The vitronectin receptor (αvβ3) was used as target to study tumor angiogenesis in vivo due to its high expression on activated endothelial and tumor cells [34].

#### 4.7.1. Biosensors/Pharmaceuticals Assessed

Two different αvβ3-targeting pharmaceutical compounds were used to image angiogenesis in xenografted mice: a radiolabeled arginylglycylaspartic acid (Arg-Gly-Asp, RGD) peptide that specifically recognizes αvβ3 integrin (^99m^Tc-RGD_2_, ININ, México), and a fluorescence imaging agent comprising both a selective, non-peptide small molecule αvβ3-antagonist, and a near-infrared (NIR) fluorochrome (IntegriSense™680). To evaluate the biosensors uptake by the tumor tissue, fluorescence, X-ray, and single-photon emission computed tomography (SPECT/CT) images were acquired in tumor-bearing mice (*n* = 2 per experimental group, for each technique). 

#### 4.7.2. Animal Preparation for Imaging

Mice were anesthetized to minimize their discomfort and movement during the biosensors administration and image acquisition. The animals were placed individually in an induction chamber, and anesthesia was supplied in a mixture of 2% isoflurane in 100% oxygen. Under anesthesia, the fluorescent and radioactive compounds were injected in the tail vein. 

#### 4.7.3. Imaging

##### Fluorescence and X-Ray Imaging

Fluorescence and X-ray images were acquired with the optical/X-ray Imaging System In-Vivo Xtreme (BVX, Bruker Corp. USA) to verify tumor uptake 24 h and 48 h after the intravenous injection of IntegriSense™680. Ex vivo images of the tumor were also acquired. Fluorescence images were obtained with an exposure time of 2 s; the binning was 4 × 4 and the field of view (FOV) was 12.5 cm. IntegriSenseTM 680 excitation and emission wavelengths were 630 and 700 nm respectively. The X-ray parameters were set as follows: energy of 45 kVp, current of 497 μA, and an aluminum filter of 0.8 mm. The exposure time was 2 s, a 1 × 1 binning, and a FOV of 12.5 cm. Using the Bruker’s MI software, the fluorescence and X-ray images were overlapped. Due to the variability of tumor morphology, the regions of interests (ROIs) were drawn freehand and quantified in terms of mean photons/s/mm^2^. 

##### SPECT/CT Imaging

Intravenous injection of ^99m^Tc-RGD_2_ (7.4 MBq in 0.05 mL) was applied to each isoflurane-anesthetized mouse. To verify the radiopharmaceutical tumor uptake, animals were individually placed in prone position inside the imaging chamber of the micro-SPECT/CT bed. SPECT and radiographic computed tomography (CT) images were acquired 3–4 h after the radiopharmaceutical injection using a micro-SPECT/CT scanner (Albira, ONCOVISION; Gem Imaging S.A., Valencia, Spain). The micro-SPECT FOV was 80 mm; a symmetric 20% window was set at 140 keV, and multipinhole collimators were used to acquire a three-dimensional SPECT image with a total of 64 projections of 30 s each over 360°. The image dataset was then reconstructed using the ordered-subset expectation maximization algorithm with the standard mode parameter, as provided by the manufacturer. CT parameters were 35 kV sure voltage, 700 pA current, and 600 micro-CT projections. 

### 4.8. Tumor Phenotype Characterization and Immunocytochemical Analysis 

After animals’ sacrifices, tumors were collected and immediately fixed in formaldehyde. Fixed and paraffin-embedded tissue sections were dewaxed and rehydrated using standard protocols. Antigen retrieval was accomplished by autoclaving in retriever citrate solution (BioSB, Santa Barbara, CA, USA). Slides were blocked with immunodetector peroxidase blocker (BioSB). The following primary antibodies were incubated for 1 h: Anti-Integrin β3 (Itgb3 1:100, Cell Signaling Technology, Beverly, MA 13166, USA), anti-ER (1:150, BioSB), anti-PR (1:150, BioSB), anti-HER2 (1:11000, BioSB), anti-CK-7 (1:200, BioSB 6650), and anti-vimentin (1:100, BioSB). After washing, the slides were sequentially incubated with Immuno-Detector Biotin-Link and Immuno-Detector HRP label (BioSB) for 10 min each. Staining was completed with diaminobenzidine (DAB) and slides were counterstained with hematoxylin. To evaluate VDR and RXR expression in MBCDF-T and EA.hy926 cells, they were seeded onto sterile chamber slides and incubated for 48 h. Cells were fixed and permeabilized in fixation/permeabilization solution at 4 °C (Cytofix/Cytoperm BD Biosciences, San Diego, CA, USA). Washing steps and antibodies dilutions were performed with perm/wash solution (BD Biosciences). Slides were co-incubated overnight at 4 °C with mouse anti-VDR antibody (1:1000, Santa Cruz Biotechnology, Santa Cruz, CA, USA, D6 SC-13133X) and rabbit anti-RXR (1:1000, Santa Cruz Biotechnology ΔN197 SC-774X). Negative controls were incubated in the absence of primary antibody. After washing, goat anti-mouse-Cy3 (1:500, Life Technologies Inc., Carlsbad, CA, USA) and goat anti-rabbit-FITC (1:500, Jackson ImmunoResearch Laboratories, West Grove, PA) were co-incubated in the chamber slides for 2 h. UltraCruz mounting medium (Santa Cruz) containing 4′,6-diamidino-2-phenylindole (DAPI) was applied and slides were coverslipped. Cells were photographed with a conventional fluorescence microscope.

### 4.9. Intratumor Vessel Density Evaluation

To further assess tumor angiogenesis, we used the method described by Weidner et al. [56]. Microvessel count was performed by three different observers using Itgb3 in photographs acquired at 20× magnification. We chose Itgb3 since it is a marker of activated endothelium. Each observer counted the number of immunostained vessels in at least three hot spots areas of each tumor. Hot spots are the densest vascularized areas within the tissue in a given field. Three hot spots per tumor were counted in at least 10 different tumors per treatment. Microvessel density was expressed as the number of Itgb3-positive tubular structures counted per hot spot field.

### 4.10. Real Time PCR Amplifications

We used the gene expression of mouse renal *Cyp27b1* and *Cyp24a1* as well as hepatic *Cyp3a44* and Glutathione peroxidase (*GPx1*) as controls for the compounds bioactivity. Total RNA was extracted from tissues using Trizol^®^ reagent (Life Technologies). The concentration of RNA was estimated spectrophotometrically at 260/280 nm and a constant amount of RNA (2 μg) was reverse transcribed using a commercial kit (Roche Applied Science, Indianapolis, IN, USA). Gene expression of the housekeeping gene β-actin (*Actb*) was used as internal control. Sequences of the upper and lower primers pairs were (5′–3′): *Cyp24a1* (NM_009996.3) aactgtacgctgctgtcacg – aatccacatcaagctgttgc; *Cyp27b1* (NM_010009.2) gagttttacaaatttggcctagaaa – gtgtcaggagggacttcagc; *Actb* (M12481.1) aaggccaaccgtgaaaagat – gtggtacgaccagaggcatac; *GPx1* (NM_008160.6) gtttcccgtgcaatcagttc – caggtcggacgtacttgagg; *Cyp3a44* (NM_177380.3) actcaccctgatatccagaagaa – agtatcacaggtgggagttgc. Corresponding probe numbers from the universal probe library (Roche) were: 78, 3, 56, 2, and 13 for *Cyp24a1*, *Cyp27b1*, *Actb*, *GPx1*, and *Cyp3a44*, respectively. Real time PCR amplifications were performed using a LightCycler^®^ 480 Instrument (Roche) and the following PCR conditions: activation of Taq DNA polymerase and DNA denaturation at 95 °C for 10 min, proceeded by 45 amplification cycles of 10 s at 95 °C, 30 s at 60 °C, and 1 s at 72 °C.

### 4.11. In Vitro Wound Healing Assay

To study the effect of the different treatments on endothelial cell migration, a wound healing assay was performed as previously described [57]. Briefly: EA.hy926 cells were seeded in 6-well plates and incubated until a confluent cell monolayer was formed (80–90%). To inhibit cell proliferation, cells were serum-starved for 24 h and then a linear thin scratch (wound) was created by using a sterile 10 μL pipette tip. The cell monolayer was then washed to remove cell debris and serum-free medium containing the treatments was added in triplicate. The plates were incubated under standard cell culture conditions for 24 h. During this time, digital photographs of the gap were acquired using bright-field microscopy (three images per well per time point: 0, 6, 12, 24 h). Wound area was calculated afterwards by using the ImageJ software. The migration rate was expressed as the change in the wound area over time. The scratch area at time point 0 h was set to 1.

### 4.12. Statistical Analysis

Statistical differences were determined by one-way ANOVA followed by the post hoc test Holm–Sidak for multiple comparisons. Comparisons between two groups were made by unpaired Student’s *t* test (SigmaStat 3.5, Jandel Scientific, San Jose, CA, USA). Differences were considered statistically significant at *p* < 0.05.

## 5. Conclusions

- Curcumin potentiated calcitriol antitumorigenic in vivo effects in TNBC by preventing intratumor vessel formation and favoring an antioxidant state.
- Curcumin antiangiogenic effect was mediated by the inhibition of endothelial cells proliferation and integrin expression.
- Resveratrol induced endothelial cell death in vitro, while in vivo inhibited angiogenesis in TNBC tumors only when combined with calcitriol.
- Curcumin solubilization in ethanol promoted its dilution in water, facilitating its administration and improving its absorption and bioavailability.
- Calcitriol coadministered with curcumin represents a promising adjuvant therapy for TNBC by inhibiting tumor growth and angiogenesis.
- Given that calcitriol and its combination with curcumin or resveratrol differentially regulated hepatic *Cyp3a44* gene expression, potential pharmacokinetic interactions should be considered when administering these compounds with CYP3A4 substrates in the clinic (including drugs and dietary compounds).

## Figures and Tables

**Table 1:** Inhibitory concentrations values at 50% (IC_50_).

Treatment	MBCDF-T	EA.hy926
Cal	1.04 nM	NA
Cur	7.96 μM	8.42 μM
Rsv	7.83 μM	8.74 μM

Half maximal inhibitory concentration of calcitriol (Cal), curcumin (Cur), and resveratrol (Rsv). NA: not applicable.

## Supplementary Materials

The following are available online at https://www.mdpi.com/2072-6694/11/11/1739/s1, Figure S1: Characterization of MBCDF-T tumors.
