# Supplementary material for: Synergistic Antitumorigenic Activity of Calcitriol with Curcumin or Resveratrol is Mediated by Angiogenesis Inhibition in Triple Negative Breast Cancer Xenografts
Source: Cancers (Basel). 2019 Nov 6;11(11):1739. doi: 10.3390/cancers11111739 (PMC6896056; doi:10.3390/cancers11111739)
Supplement: Supplementary file 1 [file cancers-11-01739-s001.pdf]

Supplementary Materials: Synergistic antitumorigenic activity of calcitriol with curcumin or resveratrol is mediated by angiogenesis inhibition in triple negative breast cancer xenografts

Janice García-Quiroz, Rocío García-Becerra, Clara Santos-Cuevas, Gerardo J. Ramírez-Nava, Gabriela Morales-Guadarrama, Nohemí Cárdenas-Ochoa, Mariana Segovia-Mendoza, Heriberto Prado-Garcia, David Ordaz-Rosado, Euclides Avila, Andrea Olmos-Ortiz, Sofía López-Cisneros, Fernando Larrea and Lorenza Díaz

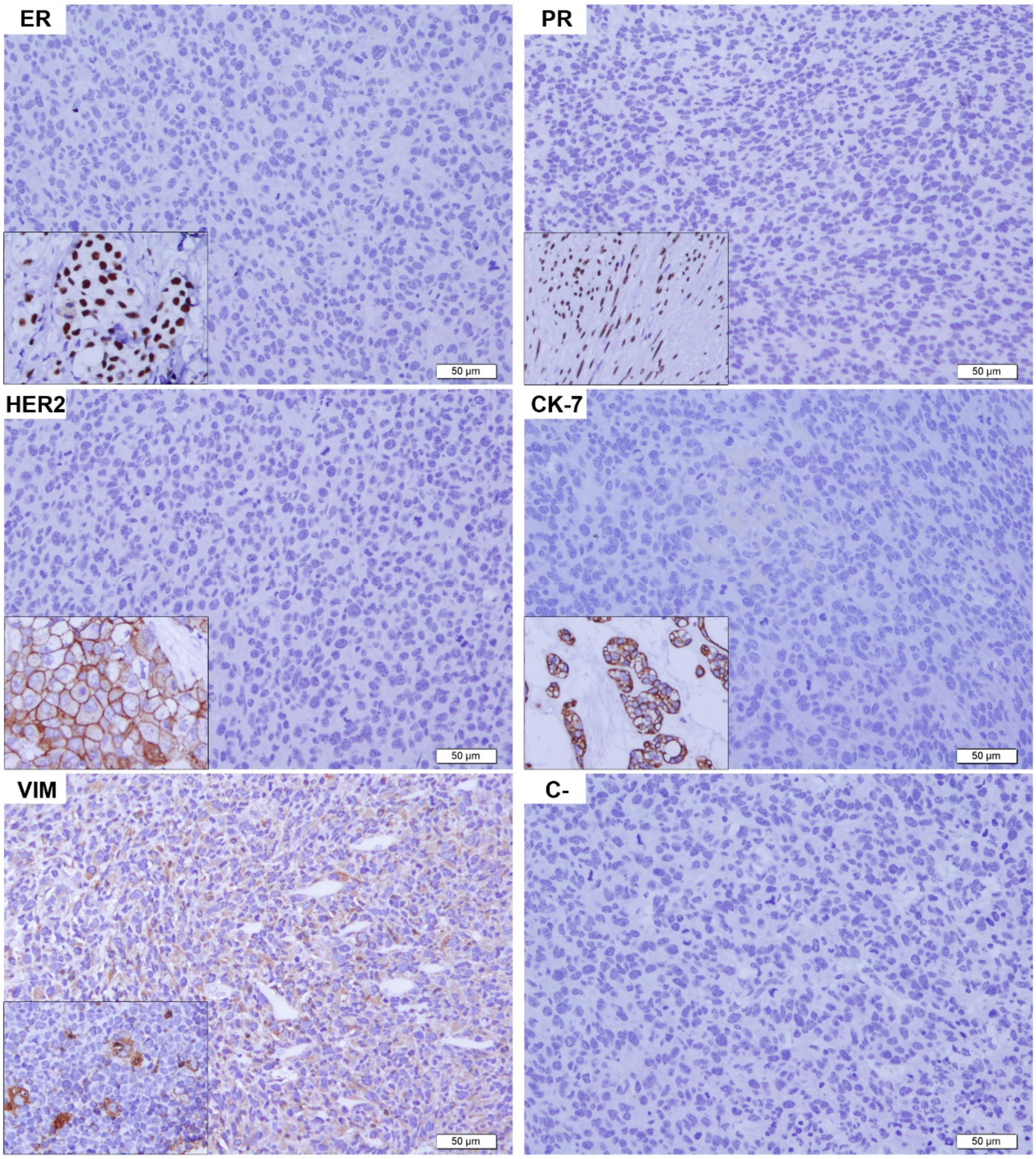

Figure S1. Characterization of MBCDF-T tumor phenotype. Breast cancer cells derived from an invasive ductal breast carcinoma were xenografted in nude mice. After approximately one month, the tumors were excised and processed for immunohistochemistry. Expression of estrogen receptor (ER), progesterone receptor (PR), Epidermal growth factor receptor 2 (HER2), cytokeratin-7 (CK-7) and vimentin (VIM) were evaluated. Positivity is shown as brown staining only in the vimentin marker. Insets show positive controls for the antibodies. Negative control is shown (C-). Representative images are provided (20 ×).
